# Ecological niche modeling of mosquito vectors of West Nile virus in St. John’s County, Florida, USA

**DOI:** 10.1186/s13071-016-1646-7

**Published:** 2016-06-29

**Authors:** Mohamed F. Sallam, Rui-De Xue, Roberto M. Pereira, Phillip G. Koehler

**Affiliations:** University of Florida, Gainesville, Florida 32611 USA; Anastasia Mosquito Control District, St. Augustine, FL 32080 USA; College of Science, Ain Shams University, Cairo, 11566 Egypt

**Keywords:** West Nile virus, *Culex nigripalpus*, *Culex quinquefasciatus*, Habitat suitability, Florida

## Abstract

**Background:**

The lack of available vaccines and consistent sporadic transmission of WNV justify the need for mosquito vector control and prediction of their geographic distribution. However, the distribution of WNV transmission is dependent on the mosquito vector and the ecological requirements, which vary from one place to another.

**Methods:**

Presence/density data of two WNV mosquito vectors, *Culex nigripalpus* and *Cx. quinquefasciatus*, was extracted within 5 km buffer zones around seropositive records of sentinel chickens in order to delineate their predicting variables and model the habitat suitability of probable infective mosquito using MaxEnt software. Different correlations between density data of the extracted mosquito vectors and 27 climate, land use-land cover, and land surface terrain variables were analyzed using linear regression analysis. Accordingly, the correlated predicting variables were used in building up habitat suitability model for the occurrence records of both mosquito vectors using MaxEnt.

**Results:**

The density of both WNV mosquito vectors showed variation in their ecological requirements. Eight predicting variables, out of 27, had significant influence on density of *Cx. nigripalpus*. Precipitation of driest months was shown to be the best predicting variable for the density of this vector (*R*^2^ = 41.70). Whereas, two variables were proven to predict the distribution of *Cx. quinquefasciatus* density. Vegetation showed the maximum predicting gain to the density of this mosquito vector (*R*^2^ = 15.74), where nestling birds, in particular exotics, are found. Moreover, Jackknife analysis in MaxEnt demonstrated that urbanization and vegetation data layers significantly contribute in predicting habitat suitability of *Cx. nigripalpus* and *Cx. quinquefasciatus* occurrence, respectively, which justifies the contribution of the former in urban and the latter in epizootic transmission cycles of WNV. In addition, habitat suitability risk maps were produced for both vectors in response to their predicting variables.

**Conclusions:**

For the first time in the study area, a quantitative relationship between 27 predicting variables and two WNV mosquito vectors within their foraging habitats was highlighted at the local scale. Accordingly, the predicting variables were used to produce a practical distribution map of probable infective mosquito vectors. This substantially helps in determining where suitable habitats are found. This will potentially help in designing target surveillance and control programmes, saving money, time and man-power. However, the suitability risk maps should be updated when serological and entomological data updates are available.

## Background

West Nile virus (WNV) disease was reported for the first time in the United States in New York City during 1999 [1] with a severe mortality of some wild native birds, in particular American crows, and many species of exotic birds [2]. Since that time, increased public health concerns have surfaced over mosquito-borne diseases due to their consistent and sporadic autochthonous transmission in the state of Florida. Eventually, WNV spread to St. John’s County, FL by 2001 [3]. Details concerning the nature of transmission dynamic and predicting variables of WNV disease in the United States remain controversial, especially with the continuous change in global climate and land use-land cover (LULC) including urban expansion and human population growth. Moreover, mosquito vector biology and their ecological requirements play a major role in the variation of the transmission cycle of WNV.

Both *Culex nigripalpus* Theobald and *Cx. quinquefasciatus* Say were recorded as the main vectors of WNV in the state of Florida [4]. The former mosquito species was incriminated in the urban transmission cycle of the virus in Peninsular Florida with a feeding preference on human blood [5–8], whereas the latter species was reported to be responsible for an epizootic cycle and sustaining the virus circulation within reservoir host bird(s) [5, 9]. However, *Cx. quinquefasciatus* alongside with *Cx. pipiens* complex, were incriminated in the urban transmission cycle of WNV in northeastern and central parts of the USA [10–12]. The biology and ecology of WNV mosquito vectors may show some elasticity from one place to another based on the available resources in the surrounding environments. Accordingly, the transmission cycle of WNV may show some variations in response to the biology and ecology of these mosquito vectors.

Surveillance and control programmes of mosquito vectors are the most effective tools for arbovirus disease prevention [13]. However, these programmes have low priority and lack adequate funds [14]. Prediction models of suitable habitats for arbovirus transmission in terms of mosquito vector distribution and seropositive sentinel chickens could maximize the potentiality of surveillance and control programmes to break the transmission cycle of WNV. Moreover, the lack of available potential vaccines for WNV and consistent development of insecticide resistance for mosquito vectors justify the necessity to model arbovirus disease so that host spot areas can be targeted during surveillance and control activities.

The previous models of WNV transmission dynamics are based solely on hydrological and meteorological data [5, 9, 15–17]. In addition, other models have been based on socio-environmental predictors in terms of vegetation or urban and sub-urban areas [18]. These previous studies addressed three major mosquito vectors in WNV transmission, *Cx. pipiens*, *Cx. quinquefasciatus,* and *Cx. nigripalpus*. The feeding preference of these mosquitoes may show significant variation from one place to another, consequently the disease transmission cycle may vary. Although these models are useful, their findings did not adequately account for the interrelationships between climate and non-climate variables such as LULC and Digital Elevation Models (DEM) and the overall influence of these interrelationships on arbovirus transmission cycles [19]. In addition, some of them either predicted the distribution risk of WNV on regional scale or used data points of WNV seropositive and mosquito vectors instead of region representing the flight range of the mosquito vectors.

*Culex nigripalpus* and *Cx. quinquefasciatus* may share some ecological habitats, but their biological and ecological requirements in terms of blood meal source, water habitat quality, vegetation type, and human activities may show significant variation. Distribution models that include climate, LULC, and DEM variables may give detailed information on variation of suitable habitats between both mosquito vectors and the virus transmission dynamics.

To address the limitations of previous studies, we developed a habitat suitability model for probable infective WNV mosquito vectors within their flight range using data records on mosquito vector occurrence and WNV seropositive sentinel chickens. In addition, we addressed the interrelationships between climate, LULC and DEM variables and their overall influence on the habitat suitability of probable infective mosquito vectors using the Maximum Entropy (MaxEnt) tool. The current study was conducted in St. John’s County, Fl. A total of 43 mosquito species were previously reported inhabiting a great diversity of salt and fresh water habitats throughout the County [20, 21]. Eleven of these mosquito species are known vectors of pathogens, notably arthropod-borne viruses (arboviruses) [3, 4, 22, 23]. The Anastasia Mosquito Control District (AMCD) oversees the entire County’s mosquito surveillance and control programmes to alleviate the risk of mosquito-borne disease transmission. Since the local economy is primarily driven by the tourism industry, the management of mosquito-borne diseases has a significant economic impact. The occurrence of arbovirus cases has a negative impact on tourism, making AMCD surveillance and control programmes an important element to the economic health of the county.

## Methods

### Study area

St. John’s County is located in northeastern Florida, USA, with a total area of 1588 km^2^ intermingled between the St. Johns River to the west and the Atlantic Ocean to the east.

### Data layers

#### Arbovirus data

Sixty chickens (21-week old) were distributed over 12 locations in the county from the beginning of April through December every year during 2007–2013 to monitor arbovirus activity. Approximately 2.0 ml of blood was taken from each chicken’s wing vein once a week. Blood samples were kept in labeled vacutainers (Fisher Scientific®) and transported back to the laboratory at the AMCD Base Station in St. Augustine Beach where they were centrifuged at 4375 RPM for 15 minutes. Samples were then placed in a labeled and sealed plastic bag, shipped to the Florida State Department of Health (FDOH) Virus Laboratory in Tampa, Florida and tested for WNV. Samples were sent on the first operation day of the week and results reported by the end of the same week. Once a chicken seroconverted, it was removed, destroyed, and replaced with a new one at its respective location.

**Fig. 1:**
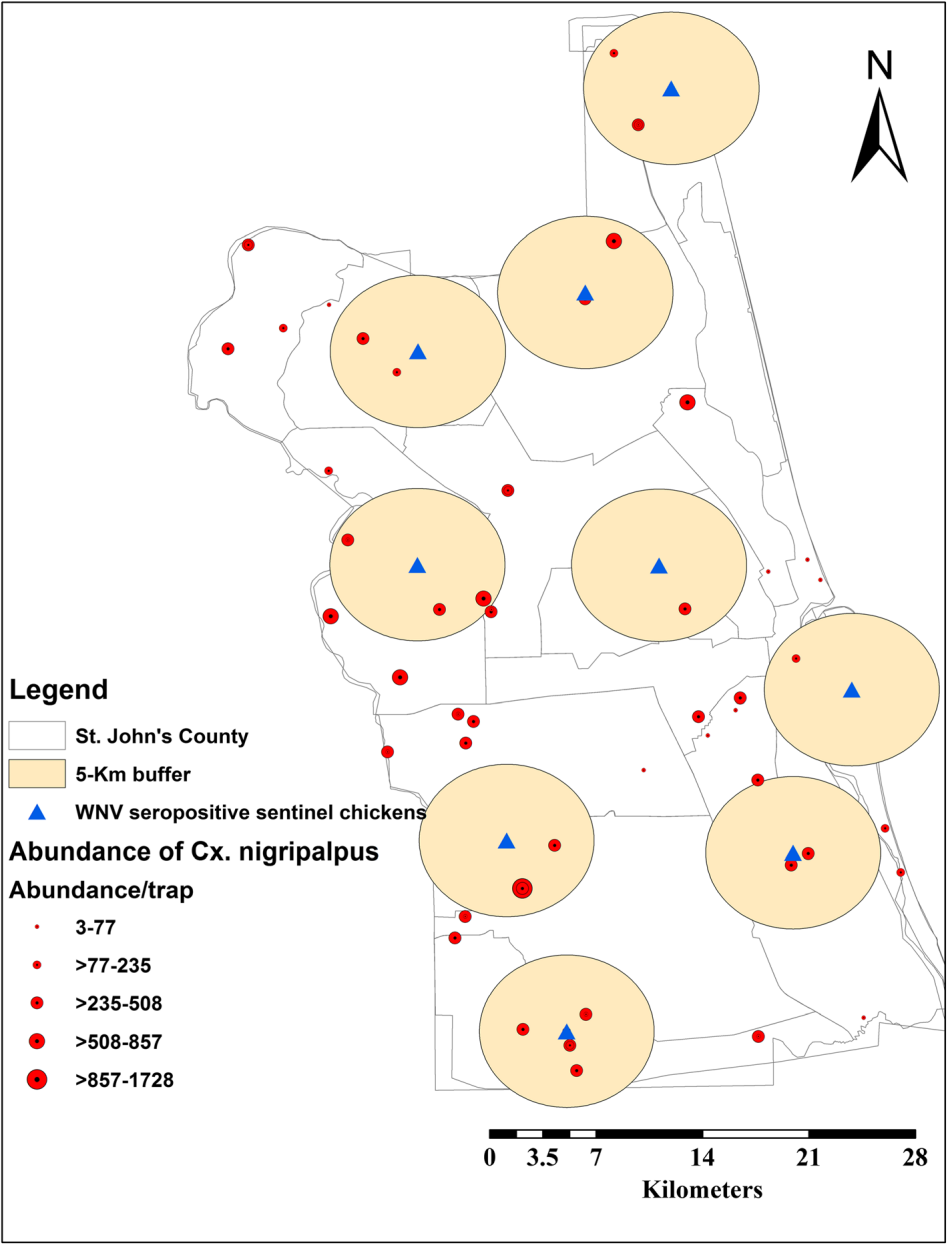
*Culex nigripalpus* sampling sites within 5 km buffer zones around WNV seropositive records in St. John’s County

**Fig. 2:**
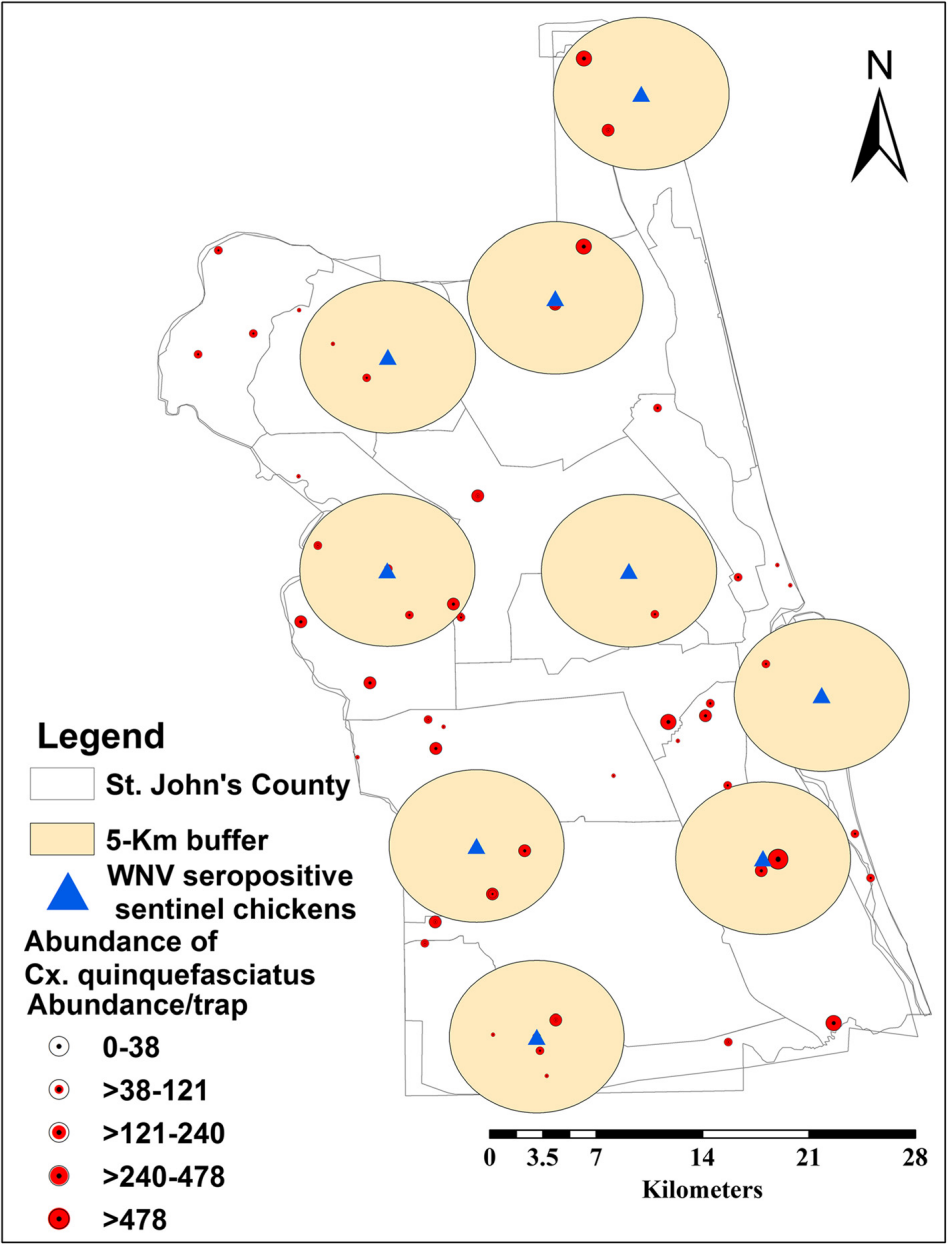
*Culex quinquefasciatus* sampling sites within 5 km buffer zones around WNV seropositive records in St. John’s County

A 5 km buffer zone around positive seroconversions was generated using a geoprocessing toolbox in ArcGIS (ver. 10.0) (Figs. 1 and 2) [24]. The buffer zones were used to extract mosquito vector sampling data (occurrence/density) as an indication of probable infective mosquito bites within their flight range around positive seroconverstions [25, 26]. Although these seropositive sentinel chickens were not included in building up the model, the buffer zones around them were used to: (i) extract the mosquito sampling points within these zones, which reflects the presence of probable infective mosquitoes, and (ii) predict distribution of probable infective mosquito as a bias file.

#### Mosquito sampling data

Population density (total number of mosquito vector/season/5 km) of adult host seeking mosquito vectors were monitored by AMCD using CDC light traps (John W. Hock Company, Gainesville, FL) baited with dry ice (BioSensory, Inc.) at 68 permanent locations in the County. Traps were placed outdoors at the beginning of March through the last week of November during 2007–2013. Traps were suspended 1 m above ground surface by a shepherd’s hook and operated for 18–20 h using a 12 V battery. Mosquito collections were transported weekly from the field to the AMCD facility for further identification to species level using the taxonomic keys of Darsie & Ward [27].

#### Bioclimate data

To predict the habitat suitability of WNV mosquito vectors, 19 bioclimatic variables (11 layers of temperature and 8 precipitation indices) and elevation layers were obtained from the WorldClim database ver.1.4 [28] (Table 1). These layers are available at a 30 arc-seconds (~1 km) resolution. The layers were clipped to match dimensions of the county and saved as ASCII grids using Model Builder in ArcGIS.

**Table 1:** Twenty seven variables used in predicting suitable habitats of WNV mosquito vectors in St. John’s County, Fl

Variable	Variable name	Data source	% Contribution
Alt	Elevation in meters	WorldClim^c^	Not included
Aspect	Aspect ratio	Generated^d^	Not included
Bio1	Annual Mean Temperature	WorldClim^c^	Not included
Bio10	Mean Temperature of Warmest Quarter	WorldClim^c^	Not included
Bio11	Mean Temperature of Coldest Quarter	WorldClim^c^	16.6^a^
Bio12	Annual Precipitation	WorldClim^c^	14.1^a^
Bio13	Precipitation of Wettest Month	WorldClim^c^	13.6^a^
Bio14	Precipitation of Driest Month	WorldClim^c^	1.9^a^
Bio15	Precipitation Seasonality (Coefficient of Variation)	WorldClim^c^	Not included
Bio16	Precipitation of Wettest Quarter	WorldClim^c^	Not included
Bio17	Precipitation of Driest Quarter	WorldClim^c^	Not included
Bio18	Precipitation of Warmest Quarter	WorldClim^c^	Not included
Bio19	Precipitation of Coldest Quarter	WorldClim^c^	Not included
Bio2	Mean Diurnal Range (Mean of monthly (max temp - min temp))	WorldClim^c^	1.5^b^
Bio3	Isothermality (BIO2/BIO7) (* 100)	WorldClim^c^	13.5^a^
Bio4	Temperature Seasonality (standard deviation *100)	WorldClim^c^	2.4^a^
Bio5	Max Temperature of Warmest Month	WorldClim^c^	Not included
Bio6	Min Temperature of Coldest Month	WorldClim^c^	Not included
Bio7	Temperature Annual Range (BIO5-BIO6)	WorldClim^c^	Not included
Bio8	Mean Temperature of Wettest Quarter	WorldClim^c^	Not included
Bio9	Mean Temperature of Driest Quarter	WorldClim^c^	Not included
Curvature	Curvature	Generated^d^	Not included
Hill shade	Hill shade	Generated^d^	12.4^a^
LAI	Leaf Area Index	MODIS^e^	98.5^b^
Slope	Slope	Generated^d^	Not included
Surface water	Lakes/ponds/streams	USGS^f^	Not included
Urbanization	Human population settlements	USGS^f^	25.5^a^

^a^Predicting variables for *Culex nigripalpus*, using linear regression analysis

^b^Predicting variables for *Culex quinquefasciatus*, using linear regression analysis

^c^WorldClim Global Climate database v1.4, available at: http://www.worldclim.org/(accessed 7/3/2015).

^d^Digital elevation model using the surface spatial analyst tool in Arc tool box of ArcGIS ver. 10.1.

^e^Moderate Resolution Imaging Spectrometer (MODIS), available at: https://lpdaac.usgs.gov/(accessed 7/3/2015)

^f^USGS available at: http://water.usgs.gov/GIS/dsdl/ds240/(accessed 7/3/2015)

All layers of variables data used in producing species distribution model gridded to ~1 Km spatial resolution and projected into the MODIS sinusoidal projection

Slope measures the rate of changes of elevation at surface location, expressed as an angle from 0 (flat) to 90° (high elevation). Aspect ratio indicates the orientation of the slope, eventually this ratio reflects the places of water accumulation and larval mosquito establishment [29, 30]. Aspect ratio ranges from 0 to 360°, however, in this study, aspect ratio was cosine-transformed to represent ranges from +1 (north-facing slope) to −1 (south-facing slope) [31]. Curvature and hill shade reflect depressions and sunlight intensity in/on land surface. The four indicators were generated from a 30 arc-seconds Digital Elevation Model (DEM).

#### Land use - land cover data

Because the distribution of mosquitoes is greatly affected by the human population as a source of blood meal [32], urban areas layer was included as a predictor for distribution of human population (Table 1). Since vegetation represents resting places and sugar meal source for adult mosquitoes, it has been extensively used as a predictor for their occurrence [33–35]. Vegetation land cover used in our model was expressed as Leaf Area Index (LAI). The LAI captures vegetation characteristics such as canopy cover and sugar resources. The LAI was derived from Moderate Resolution Image Spectroradiometer (MODIS) imagery from the Terra Satellite. The LAI is defined as the one-sided green leaf area per unit ground area in broadleaf canopies and as one half the total needle surface area per unit ground area in coniferous canopies. The LAI represented a good indicator for LULC in canopies and forest areas, such as the case in St. John’s County rather than Normalized difference vegetation index (NDVI) [36] that gives a broader depiction about the green areas. Therefore, we used LAI as a potential remote sensing indicator for vegetation cover to assess their predictive power on distribution of both WNV mosquito vectors.

The surface water has been extensively used in previous studies as an indicator for mosquito distribution range [37]. Thereby, data layers on surface water, including water bodies, rivers and streams, were also included in the linear regression model. Types of surface water were imported, classified according to their types, clipped to the buffer zones around the sentinel chickens. Each water body type was extracted as a separate ASCII file to be used in further analysis.

#### Variable selection

**Fig. 3:**
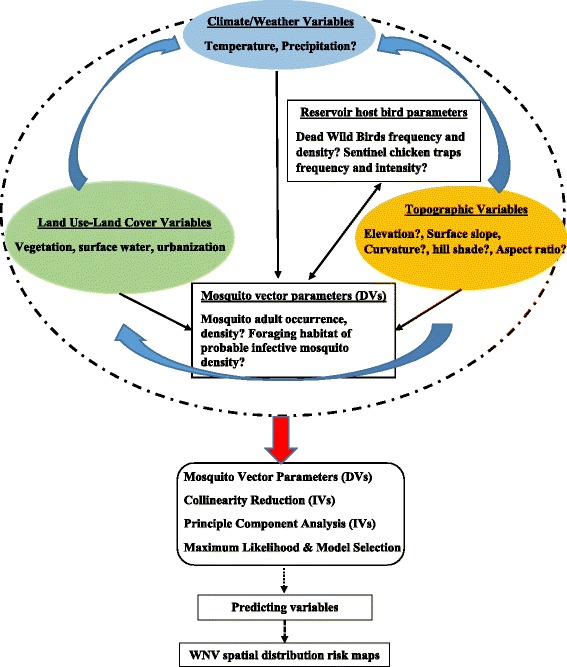
West Nile Virus transmission model, and expected outcomes in response to proposed predicting variables

A total of 27 bioclimatic, LULC and DEM data layers (Table 1) were clipped to the study site and extracted within the 5 km buffer zones around seropositive chickens in preparation for collinearity test and select variables to be included in habitat suitability model (Fig. 3). A stepwise linear regression model (LRM) was carried out to overcome redundancy and exclude the linearly correlated variables using JMP pro statistical package ver. 10.0.0 [38]. The collinearity occurs when two or more predictor variables in a model are highly correlated, so one variable can be accurately estimated from the linear relationship with other variables. This analysis was carried out to test the dependency of both mosquito vectors density on their predicting variables within the flight range of these vectors around WNV seropositive. The minimum corrected Akaike Information Criterion (AICc) value was used to select variables that potentially predicts the density of the mosquito vectors. Although data redundancy does not reduce the reliability or predictive power of the model, it may influence the calculations regarding the contribution percentage of individual predictors especially at the local scale studies such as used here.

### Habitat suitability modeling of WNV vectors

The MaxEnt software v. 3.3 [39–41] was used to model the suitable habitats of both mosquito vectors. The software uses the occurrence (presence of mosquito vector at least one time/trap/season) records of mosquito vectors in association with the predicting variables to generate suitability risk maps.

The contribution of predicting variables in our model was evaluated using Jackknife analysis in MaxEnt. The predicted habitat probability was categorized into five classes using the natural area breaks in ArcGIS utilizing the training omission rate at 10 percentile training presence value produced from MaxEnt: very low (0–0.1), low (> 0.1–0.2), medium (> 0.2–0.4), high (> 0.4–0.6), and very high (> 0.6) using natural breaks in the symbology tools in ArcGIS.

The presence records of both mosquito vectors were randomly partitioned for model evaluation into two subsamples: 75 % of the records were used for training and building up the model, and 25 % of the records were used for testing the model’s accuracy. The duplicate records of WNV mosquito vectors within ~1 km of the same cell size were excluded [42].

Five replicate runs were assigned in running the model to generate the average, maximum, minimum and median of the distribution range of mosquito vectors. Two thresholds have been used to examine the performance accuracy of our model based on Phillips et al. [40] and Phillips & Dudik [41]: (i) the extrinsic omission was evaluated at fixed threshold (10 percentile training presence) and (ii) the area under the curve (AUC) of the receiver operating characteristics (ROC) [40, 41].

To increase the potentiality of the habitat suitability model and maximize the sampling effort of extracted mosquito vectors, a separate ASCII file generated from the 5 km buffer zones was included and weighted to the corresponding mosquito vector density [43]. Accordingly, the habitat suitability of each of the mosquito vectors will be predicted in regards to the flight range of probable infective mosquito and response to the predicting variables. Therefore, the distribution map produced for each mosquito vector reflects the presence of probable infective mosquito. The MaxEnt evaluates different correlations between the presence records of extracted mosquito vectors data and their predicting variables within the sampled areas utilizing logistic regression analysis. In our study, we weighted the presence records to density of mosquito vectors 5 km around seropositive sentinel chickens and attached as ASCII bias file. The sampling points were randomly grouped into 75 % and 25 % for data training and testing, respectively. During data training, a matrix of spatial correlations between sampling points and their associated predicting variables were created. Accordingly, the habitat suitability maps were created for sampled and unsampled areas based on the habitat similarity between sampled and unsampled regions. The suitable habitats for unsampled areas were predicted utilizing habitat similarity between sampled and unsampled ones.

## Results

### Data layers and variables selection

Density of both WNV mosquito vectors showed variation in their response to the 27 variables used in LRM. Out of 27 variables, density of *Cx. nigripalpus* demonstrated a significant correlation with eight predicting variables (AICc = 940.58, *R*^2^ = 41.70, *P* < 0.01). Three of these variables were temperature related variables namely temperature isothermality (Bio3), temperature seasonality (Bio4), and mean temperature of coldest quarter (Bio11). In addition, three precipitation related variables correlated with this mosquito vector density: the annual precipitation (Bio12) and precipitation of wettest (Bio13) and driest months (Bio14). Urbanization (*r*_(5)_ = -38.61, AICc = 943.00, *R*^2^ = 29.52, *P* < 0.05) and hill shade (*r*_(2)_ = -222.06, AICc = 945.12, *R*^2^ = 19.39, *P* < 0.01) showed significant association with the density of *Cx. nigripalpus* (Table 2). Density of this vector was found to be negatively correlated with temperature related variables, hill shade, and urbanization. However, the precipitation of driest month (Bio14) was positively correlated (*r*_(9)_ = 24.09, AICc = 940.58, *R*^2^ = 41.70, *P* < 0.05) with density of *Cx. nigripalpus* (Table 2). Whereas, *Cx. quinquefasciatus* density was found to be significantly correlated with only two variables: temperature diurnal range (Bio2) (*r*_(2)_ = -28.63, AICc = 858.29, *R*^2^ = 7.37, *P* < 0.01) and vegetation (LAI) (*r*_(3)_ = -0.93, AICc = 854.22, *R*^2^ = 15.74, *P* < 0.01). Both precipitation of driest month (Bio14) (*R*^2^ = 41.7) and LAI (*R*^2^ = 15.74) were demonstrated to be the key predictors for both *Cx. nigripalpus* and *Cx. quinquefasciatus*, respectively, in association with the other predicting factors (Table 2).

**Table 2:** Summary of stepwise linear regression analysis on density of both WNV mosquito vectors in response to bioclimatic, LULC and DEM variables

WNV vector	Variable	Coefficient	*p*	*R* ^2^	AICc
*Cx. nigripalpus*	Bio3	-63.45*	4	28.01	942.03
	Bio4	-3.46*	7	34.01	943.59
	Bio11	-159.59*	6	30.52	944.51
	Bio12	-17.74*	3	23.11	944.17
	Bio13	-2.13**	8	38.47	941.49
	Bio14	24.09*	9	41.70	940.58^a^
	Hill shade	-222.06**	2	19.39	945.12
	Urban	-38.61*	5	29.52	943.00
*Cx. quinquefasciatus*	Bio2	-28.63**	2	7.37	858.29
	LAI	-0.93**	3	15.74	854.22^a^

*P < 0.05; **P < 0.01

^a^Best predictor

### Ecological niche modeling of west Nile virus vectors

**Fig. 4:**
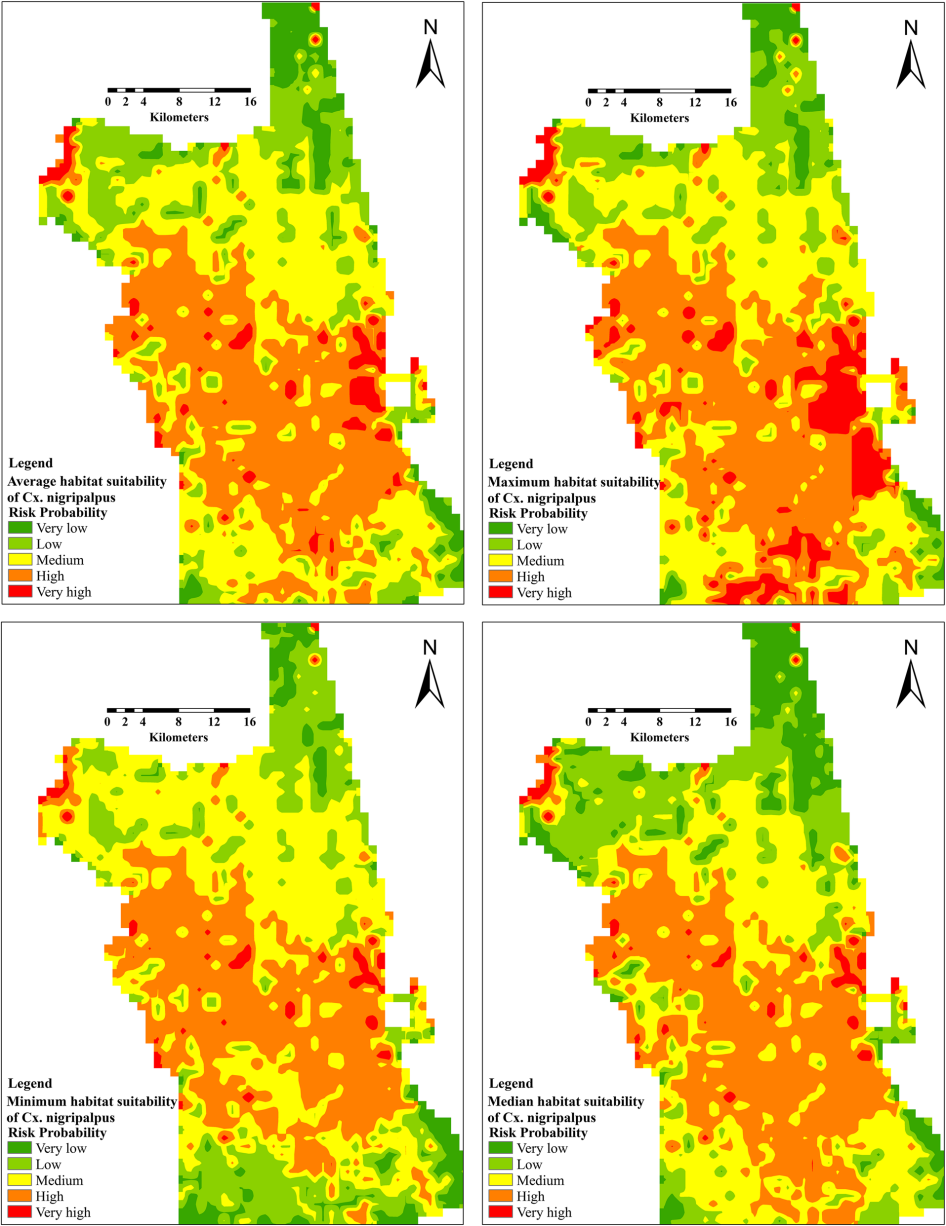
Average, maximum, minimum and median habitat suitability prediction model of *Cx. nigripalpus*

A total of 47 and 51 presence data records of *Cx. quinquefasciatus* and *Cx. nigripalpus*, respectively, out of 68 sampling points were included in habitat suitability model. For *Cx. nigripalpus*, a total of 36 and 11 sampling points were used in MaxEnt for training and testing the habitat suitability. The average predictive performance was found to be high with an AUC value of 0.75 and 0.62 for training and test occurrence records, respectively, with a standard deviation of 0.07. The fractional predicted area at 10-percentile training presence was 0.58. These points were classified as significantly better than random (*P* < 0.01). MaxEnt predicted an average of 99 km^2^ of very high suitable habitat (predicted risk probability > 0.60), which is 6.11 % of the total area of St. John’s County (Fig. 4).

**Fig. 5:**
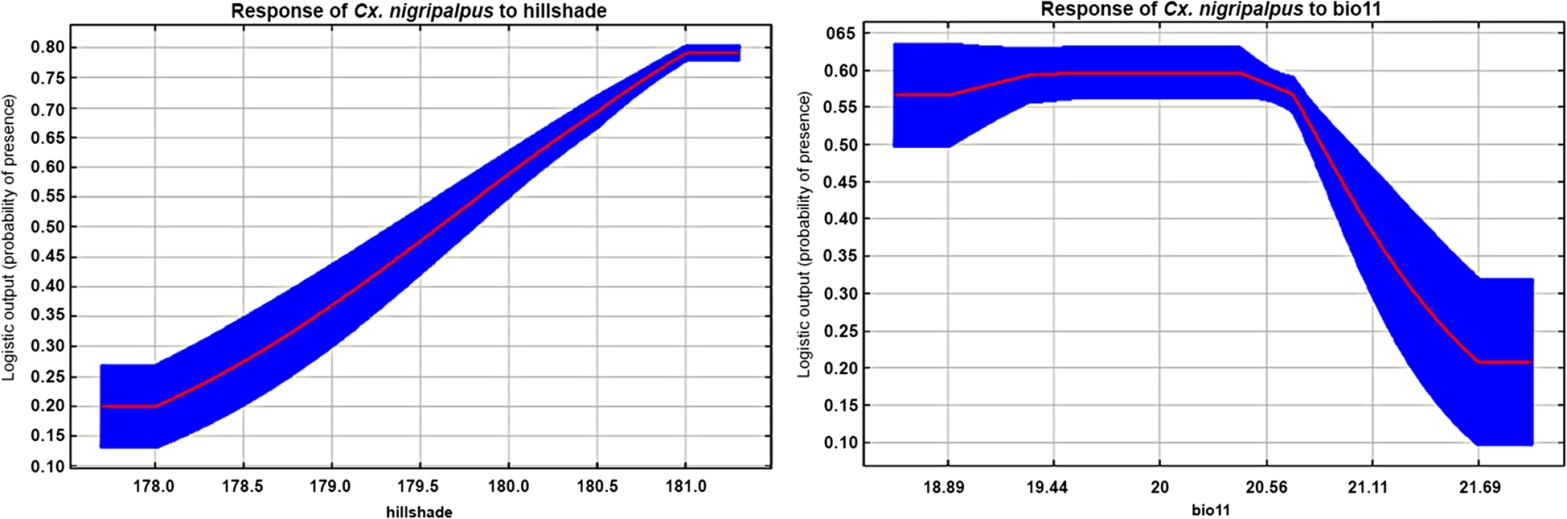
Response curve of *Cx. nigripalpus* to predicting variables in Jackknife test*. *Blue line denotes the minimum and maximum response of mosquito vector to the predicting variables

Unsurprisingly, the Jackknife test represented that temperature related variables (Bio3, 4, 11) significantly improved the predictive power by up to 32.5 %. The highest training gain was shared with precipitation related variables and urbanization (29.6 and 25.5 %). In addition, hill shade shared reduced training gain (12.4 %) in predicting suitable habitats of this mosquito vector (Table 1). Although LRM showed a negative correlation between this vector and some predicting variables such as temperature and hill shade, the maximum response of *Cx. nigripalpus* presence (> 0.5) was predicted at hill shade ranges 180–181, and temperature of the coldest quarter (Bio 11) < 21 °C with AUC training gain of 0.80 (Fig. 5). At which, the maximum likelihood of suitable habitats for the high risk area was 714 km^2^.

**Fig. 6:**
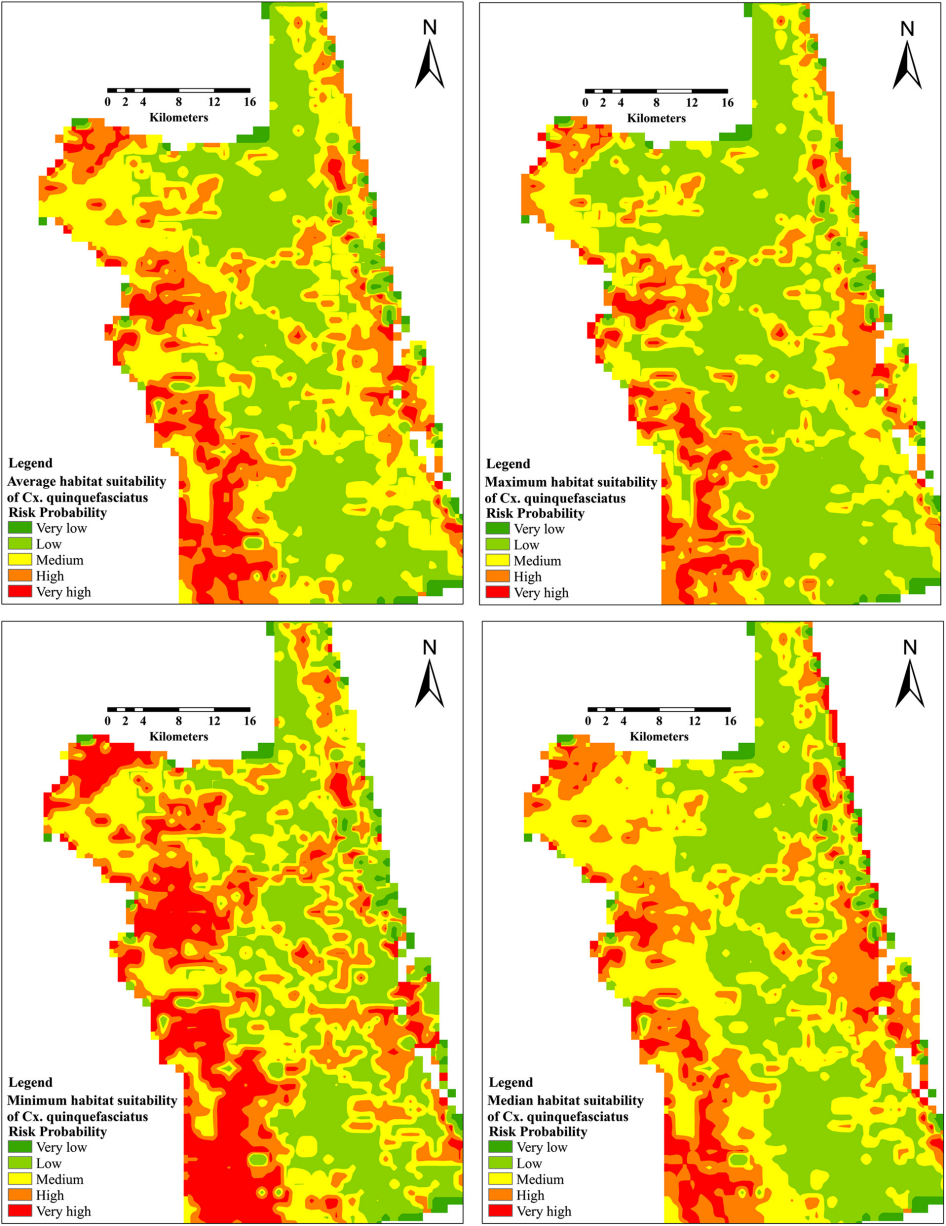
Average, maximum, minimum and median habitat suitability prediction model of *Cx. quinquefasciatus*

**Fig. 7:**
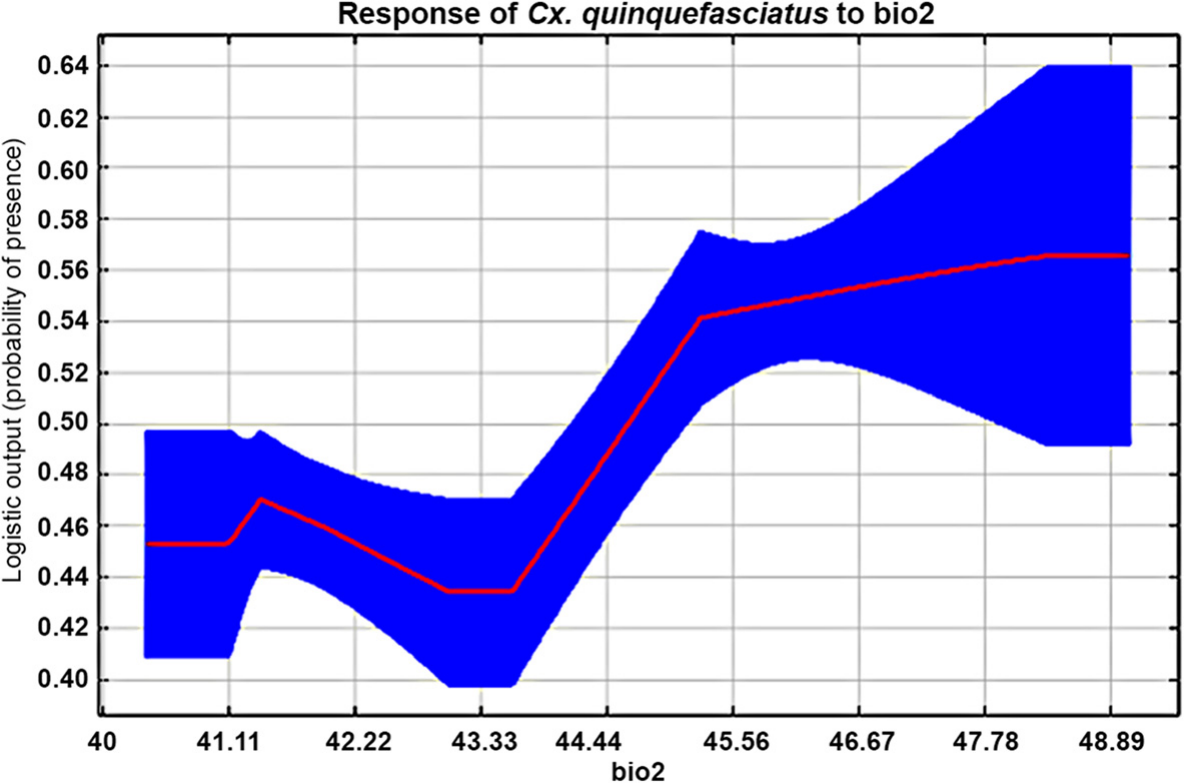
Response curve of *Cx. quinquefasciatus* to Mean Diurnal Range (Mean of monthly (max temp - min temp)) in Jackknife test*. *Blue line denotes the minimum and maximum response of mosquito vector to the predicting variables

On the other hand, a total of 39 and 12 presence points were included in training and testing of suitability model of *Cx. quinquefasciatus.* The AUC for habitat suitability model of this vector was 0.76 and 0.73 for train and test occurrence records, respectively, and a standard deviation of 0.06. The fractional predicted area at 10-percentile training presence was 0.55. The predicted average area with very high suitable habitats was 244 km^2^ (predicted risk probability > 0.60), representing 15.37 % of the total area of the County (Fig. 6). The LAI significantly improved the predictive power (98.5 %) for habitat suitability of *Cx. quinquefasciatus* compared to mean diurnal temperature range (1.5 %) (Table 1, 2). The mean diurnal range is a precursor of mean monthly temperature (maximum temp. – minimum temp.). Similarly, the habitat suitability of this mosquito vector showed a negative correlation with the mean diurnal range (Table 2). However, the maximum response of this vector was predicted at Bio2 ranges > 44.4 °C (Fig. 7).

## Discussion

In the current study, we produced a practical habitat suitability map for probable infective WNV mosquito vectors in St. John’s County. We addressed limitations in previous comparable models in regards to (i) use ~1 km resolution remote sensing data layers in terms of climate, ecological, and DEM variables, (ii) ranking the average contribution of predicting variables correlated to the density and presence of WNV mosquito vectors, and (iii) production of practical distribution risk map for the probable infective mosquito vectors confined to their flight range around seropositive records at local scale to prioritize the surveillance and control activities.

Generally, the predicted habitat suitability maps showed a significant variation in distribution between both mosquito vectors. The very high suitable habitats for *Cx. nigripalpus* was sporadically distributed in the central and southwestern areas. Unlikely, the habitat suitability produced for *Cx. quinquefasciatus* was largely confined to the western areas of the county (Figs. 4 and 6). However, the predicted distribution of both mosquito vectors shared some suitable habitats in the western regions.

Since the distribution of mosquito vectors and the virus circulation are confined to areas rather than sampling points, we extracted mosquito sampling points within their 5 km flight range around WNV seropositive records. This reflects the association between a probable infective mosquito population, WNV reservoir bird host(s), and water breeding habitats [25, 26]. Both WNV mosquito vectors are well known as exophilic and exophagic and their adult breeding habitats range from ditches, woodland pools, and freshwater marshes of a semi-permanent or permanent nature. In addition, *Cx. quinquefasciatus* shows preference to polluted water ranging from agricultural drains and sewage canals. *Cx. nigripalpus* shows feeding preference for humans rather than *Cx. quinquefasciatus*, which prefers avian blood [6–9, 15–17]. However, the former vector may feed on avian blood during the dry season, when it comes in close vicinity with reservoir host bird(s) seeking water breeding habitats [9, 15–17]. The LRM demonstrated that precipitation during the wettest (*R*^2^ = 38.47) and driest (*R*^2^ = 40.70) months have a significant role in predicting *Cx. nigripalpus* density during the dry season (Table 2) [5, 22]. Accordingly, MaxEnt confirmed this finding and demonstrated the significant contribution of urbanization (25.5 %) (Table 1) in predicting *Cx. nigripalpus*, which reflects the importance of this mosquito vector in the urban transmission cycle rather than *Cx. quinquefasciatus* [5–7, 16].

Although no WNV human cases were recorded during the study period, the transmission potential still exists due to the availability of competent mosquito vectors, reservoir hosts, probably exotic birds, and suitable ecological habitats. In addition, the lack of data on vector survival rate, feeding preference, gonotrophic cycle period, and competence for the study area prevented us including this information in building up our model. Our spatial distribution model shed light on the information gap in vector capacity parameters, which are needed to deeply understand when, precisely, transmission onset occurs in St. John’s County.

The distribution of these mosquito vectors, like other exophilic mosquitoes, are influenced by surrounding climate and ecological variables. In addition, this distribution gives deep insights on the virus circulation between the bridging and main mosquito vector, reservoir bird and human hosts. The findings of some previous models showed great potential in understanding the biology and ecology of WNV mosquito vectors in state of Florida [9, 16, 22], however, they encountered some limitations in emphasizing the interaction between ecological, climate, DEM variables and their overall influence on mosquito vector distribution at a local scale. Other models concluded the association between WNV transmission was either with forested and urban land [11, 44] or socioeconomic status [45]. However, their findings may not be applicable in other landscapes that encounter WNV transmission due to the heterogeneity in climate and landscape variables [46]. Moreover, some of the previous models predicted the spatial distribution risk of virus circulation based on sampling points rather than the flight range area of these mosquito vectors.

Unlike previous models, we tried to model the spatial distribution of probable infective mosquitoes using the mosquito presence records within their flight range around WNV seropositive records of sentinel chickens. Since arbovirus transmission is vector-density dependent, we evaluated the response of both vectors’ density to their predicting variables. The LRM provides reliable information on the influence of the predicting variables on mosquito vectors density. In this regard, the predicting variables were evaluated and selected using AICc values. Statistical LRM potentially resolves the significant discrepancy in predicting variables for both mosquito vectors. This discrepancy reflects variation in ecological requirements between both mosquito species. The potentiality of the current prediction model was proven by the high AUC values produced by MaxEnt, indicating that occurrence records were likely to be assigned a higher probability of presence than background sites.

Unsurprisingly, temperature and precipitation related variables significantly increased the prediction gain of probable infective *Cx. nigripalpus* in regards to their density and presence records. Their contribution was demonstrated by LRM and MaxEnt tools in terms of *R*^2^ values and percent contribution, respectively. The contribution of precipitation in driest (*R*^2^ = 41.70, 1.9 %) and wettest (*R*^2^ = 38.47, 13.6 %) months reflects their significance in determining the habitat suitability for this mosquito vector during dry and wet seasons. The shared high predictive power by temperature related variables (32.5 %) confirmed the association between this mosquito vector, reservoir hosts, and human population, especially during the dry season. The significance of urbanization in predicting *Cx. nigripalpus* demonstrates the suitability of this habitat for mosquito vector density (*R*^2^ = 29.52) and presence probability (25.5 %).

Accordingly, precipitation in the driest months, during the dry season in May – June, may play a major role in shifting breeding habitats of this mosquito and bring them in contact with WNV infected host birds [15]. Alternatively, precipitation of wettest months during July and August increases the water habitat suitability and density/occurrence probability of mosquito vectors. Eventually, this mosquito vector starts to get closer to urbanization areas again and bites human [9, 16, 17]. In addition, this may reflect the importance of this mosquito vector in the urban cycle and infection of the human population, which is confirmed by their feeding preference for humans in previous studies [5, 15, 16, 22].

The contribution of temperature in predicting density and distribution of this mosquito vector was highlighted in a mathematical simulation model by Lord & Day [22], in which the mortality of WNV mosquito vector populations at 22.5 °C was included as a determinant parameter for disease transmission. However, the number of parameters regarding mosquito biology and ecology prevented investigating different correlations in depth. In the current study, both LRM and Jackknife analysis showed these different interrelationships between *Cx. nigripalpus* and correlated variables within mosquito vector foraging habitats. The temperature of the coldest quarter (< 21 °C) contributed significantly in predicting the occurrence and spatial distribution of this mosquito vector.

Land topography and geomorphology were potentially used in predicting suitable water habitats of mosquitoes [30, 31, 47]. Four potential indicators were highlighted in previous studies to predict suitable water habitats of mosquitoes: aspect ratio, slope, land surface curvature and hill shade [29, 30, 48, 49]. These indicators give detailed information about locations where water flows and accumulates. Although we did not include data on larval mosquitoes, data on host seeking adult mosquitoes included in the current study reflects the vicinity of water habitats to collected samples. In regards to elevation, it defines soil-water gravitational potential energy [48] and surface water movement within drainage channels as well as throughout the landscape. Similarly, slope has the potential influence on surface water flow velocity, drainage and accumulation of water [29, 49].

In the present study, hill shade was used as a promising remote sensing data that reflects areas with surface water accumulation [31]. Hill shade showed a significant prediction gain with precipitation and temperature related variables. Among five land surface geomorphic predictors representing elevation, slope, aspect ratio, curvature, and hill shade, only the latter was demonstrated as a potential predicting variable in determining the occurrence/density and distribution of *Cx. nigripalpus* using LRM and MaxEnt. Hill shade was used in previous studies as a predicting precursor for water suitable habitats of malaria vectors [31].

Density of *Cx. quinquefasciatus* was evidently correlated with vegetation and mean temperature diurnal range. Vegetation is important for mosquitoes as a source of sugar meal, but also reflects nestling habitats for reservoir bird hosts, probably human hosts in rural areas. Feeding preference of this mosquito vector is reportedly varied [8]; however, the blood meal identification in fed mosquitoes revealed their feeding preference to avian blood rather than human [6–8]. Accordingly, this mosquito vector may play a major role in sustaining the epizootic cycle of WNV.

## Conclusions

Although some previous models showed potential in understanding the empirical correlations between WNV transmission and some ecological and climate variables in the state of Florida, these models encountered some limitations. These models predicted the spatial distribution risk of virus circulation based on sampling points rather than the flight range area of probable infective mosquito vectors at the local scale. Other models may not be applicable in other landscapes due to the heterogeneity in climate and environmental variables. The variation of landscape and climate variables greatly impact the biology and ecology of mosquito vectors in terms of feeding preference, habitat suitability, and the distribution of reservoir bird host(s), especially exotic birds.

In this model, we demonstrated how GIS, remote sensing data, and habitat suitability modeling tools can be used efficiently to analyze and identify suitable habitats for infective WNV mosquito vectors and assess the distribution risk of this disease at a local scale. Our model is very novel in addressing limitations of previous similar models at the local scale. The risk maps produced will effectively help in determining where suitable habitats are found. This will potentially help in targeted surveillance and control programmes, which will save money, time and man-power, especially if the suitability risk maps are updated with serological and entomological data when available.

The availability of consistent temporal data, in terms of mosquito and remote sensing, limit our current spatial model to delineate accurately, when WNV transmission can occur. Further temporal analysis is needed to couple the current spatial model with the updated data on mosquito distribution to give more detailed information on where and when the suitable habitats of WNV transmission may occur. In addition, information on vector capacity in regards to vector competence, survival rate, and gonotrophic cycle period, need to be considered in building up the spatial-temporal models. Eventually this will give deep insights on the competency of mosquito vector populations in transmitting WNV.

## Abbreviations

AICc, Corrected Akaike information criterion (AICc); AMCD, Anastasia mosquito control district; ASCII, American standard code for information interchange; AUC, area under curve; Bioclim, bioclimate variables; Cx, culex; DEM, digital elevation model; FDOH, Florida Department of Health; hrs, hours; km, kilometer; LAI, leaf area index; LRM, linear regression model; LULC, land use - land cover; MaxEnt, maximum entropy; MODIS, Moderate Resolution Image Spectroradiometer (MODIS); NDVI, Normalized difference vegetation index (NDVI); ROC, receiver operating characteristics; WNV, West Nile Virus

## References

[1] Centers for Disease Control and Prevention (2000). Guidelines for surveillance, prevention and control of West Nile virus infection—United States. MMWR.

[2] Steele KE, Linn MJ, Schoepp RJ, Komar N, Geisbert TW, Manduca RM (2000). Pathology of fatal West Nile virus infections in native and exotic birds during the 1999 outbreak in New York City, New York. Vet Pathol. Online.

[3] Blackmore CG, Stark LM, Jeter WC, Oliveri RL, Brooks RG, Conti LA (2003). Surveillance results from the first West Nile virus transmission season in Florida, 2001. Am J Trop Med Hyg.

[4] Sardelis MR, Turell MJ, Dohm DJ, O’Guinn ML (2001). Vector competence of selected North American *Culex* and *Coquillettidia* mosquitoes for West Nile virus. Emerg Infect Dis.

[5] Day JF (2001). Predicting St. Louis encephalitis virus epidemics: lessons from recent, and not so recent, outbreaks. An Rev Entomol.

[6] Kilpatrick AM, Daszak P, Jones MJ, Marra PP, Kramer LD (2006). Host heterogeneity dominates West Nile virus transmission. Proc R Soc Biol..

[7] Molaei G, Andreadis TG, Armstrong PM, Bueno R, Dennett JA, Real SV (2007). Host feeding pattern of *Culex quinquefasciatus* (Diptera: Culicidae) and is role in transmission of West Nile Virus in Harris County. Texas. Am J Trop Med Hyg..

[8] Turell MJ, Spring AR, Miller MK, Cannon CE (2002). Effect of holding conditions on the detection of West Nile viral RNA by reverse transcriptase-polymerase chain reaction from mosquito (Diptera: Culicidae) pools. J Med Entomol.

[9] Shaman J, Stieglitz M, Stark C, Le Blancq S, Cane M (2002). Using a dynamic hydrology model to predict mosquito abundances in flood and swamp water. Emerg Infect Dis.

[10] Brown H, Diuk-Wasser M, Andreadis T, Fish D (2008). Remotely-sensed vegetation indices identify mosquito clusters of West Nile Virus vectors in an urban landscape in the Northeastern United States. Vector Borne Zoonotic Dis.

[11] Brown HE, Childs JE, Diuk-Wasser MA, Fish D (2008). Ecological factors associated with West Nile virus transmission, Northeastern United States. Emerg Infect Dis..

[12] DeGroote JP, Sugumaran R, Brend SM, Tucker BJ, Bartholomay LC (2008). Landscape, demographic, entomological, and climatic associations with human disease incidence of West Nile virus in the state of Iowa, USA. Int J Health Geogr..

[13] Eldridge BF (1987). Strategies for surveillance, prevention, and control of arbovirus diseases in western North America. Am J Trop Med Hyg..

[14] Gubler DJ (2002). The global emergence/resurgence of arboviral diseases as public health problems. Arch Med Res..

[15] Day JF, Shaman J (2008). Using hydrologic conditions to track the risk of focal and epidemic arboviral transmission in peninsular Florida. J Med Entomol..

[16] Shaman J, Day JF, Stieglitz M (2002). Drought-induced amplification of Saint Louis encephalitis virus, Florida. Emerg Infect Dis..

[17] Shaman J, Day JF, Stieglitz M (2003). St. Louis encephalitis virus in wild birds during the 1990 south Florida epidemic: the importance of drought, wetting conditions, and the emergence of *Culex nigripalpus* (Diptera: Culicidae) to arboviral amplification and transmission. J Med Entomol.

[18] Naish S, Mengersen K, Hu W, Tong S (2013). Forecasting the future risk of Barmah forest virus disease under climate change scenarios in Queensland, Australia. PLoS ONE..

[19] Rogers D, Randolph S (2000). The global spread of malaria in a future, warmer world. Science..

[20] AMCD (2012). About AMCD operations.

[21] Naranjo D, Qualls W, Jurado H, Perez J, Xue R-D, Gomez E (2014). Vector control programs in Saint Johns County, Florida and Guayas, Ecuador: successes and barriers to integrated vector management. BMC Public Health.

[22] Lord CC, Day JF. Simulation studies of St. Louis Encephalitis and West Nile Viruses: The impact of bird mortality. Vector Borne Zoonotic Dis. 2001;1(4):317–29. 10.1089/15303660160025930 12653130

[23] Cupp EW, Klingler K, Hassan HK, Viguers LM, Unnasch TR (2003). Transmission of Eastern Equine Encephalomyelitis virus in Central Alabama. Am J Trop Med Hyg.

[24] ESRI (2011). ArcGIS desktop: release 10.

[25] DeMeillon B (1934). Studies on insects of medical importance in South Africa. South African Inst Med Res..

[26] Nayar JK, Sauerman DM (1973). A comparative study of flight performance and fuel utilization as a function of age in females of Florida mosquitoes. J Insect Physiol.

[27] Darsie RF, Ward RA (2005). Identification and geographical distribution of the mosquitos of North America.

[28] Hijmans RJ, Cameron SE, Parra JL, Jones PG, Jarvis A (2005). Very high resolution interpolated climate surfaces for global land areas. Int J Climatol.

[29] Gorsevski PV, Gessler P, Foltz RB (2000). Spatial prediction of landslide hazard using discriminant analysis and GIS. GIS in the Rockies 2000 Conference and Workshop Applications for the 21st Century Denver, Colorado September 25 - 27, 2000.

[30] Mushinzimana E, Munga S, Minakawa N, Li L, Feng CC, Bian L (2006). Landscape determinants and remote sensing of anopheline mosquito larval habitats in the western Kenya highlands. Malar J..

[31] Nmor JC, Sunahara T, Goto K, Futami K, Sonye G, Akweywa P (2013). Topographic models for predicting malaria vector breeding habitats: potential tools for vector control managers. Parasit Vectors..

[32] Burkett-Cadena ND, McClure CJW, Estep LK, Eubanks MD (2013). Hosts or habitats: What drives the spatial distribution of mosquitoes?. Ecosphere.

[33] Gu W, Müller G, Schlein Y, Novak RJ, Beier JC. Natural plant sugar sources of *Anopheles* mosquitoes strongly impact malaria transmission potential. PLoS One. 2011;6(1):e15996. 10.1371/journal.pone.0015996 PMC3024498 21283732

[34] Andersson IH, Jaenson TGT (1987). Nectar feeding by mosquitoes in Sweden, with special reference to *Culex pipiens* and *Cx. torrentium*. Med Vet Entomol..

[35] Foster WA (1995). Mosquito sugar feeding and reproductive energetics. An Rev Entomol..

[36] Zhu G, Ju W, Chen JM, Liu Y (2014). A Novel Moisture Adjusted Vegetation Index (MAVI) to reduce background reflectance and topographical effects on LAI retrieval. PLoS ONE.

[37] Ammar SE, Kenawy MA, Abdel-Rahman HA, Gad AM, Hamed AF. Ecology of the mosquito larvae in urban environments of Cairo Governorate, Egypt. J Egypt Soc Parasitol. 2012;42(1):191–202. 10.12816/0006307 22662608

[38] SAS Institute Inc. 2013. Using JMP 11. Cary, NC: SAS Institute Inc.

[39] Phillips SJ, Anderson RP, Schapire RE (2006). Maximum entropy modeling of species geographic distributions. Ecol Modelling.

[40] Phillips S, Dudik M, Schapire R (2004). A maximum entropy approach to species distribution modeling. Proceedings of the 21st International Conference on Machine Learning.

[41] Phillips S, Dudik M (2008). Modeling of species distributions with Maxent: new extensions and a comprehensive evaluation. Ecography..

[42] Zhou G, Munga S, Minakawa N, Githeko A, Yan G (2007). Spatial relationship between adult malaria vector abundance and environmental factors in western Kenya highlands. Am J Trop Med Hyg.

[43] Phillips SJ, Dudík M, Elith J, Graham CH, Lehmann A, Leathwick J (2009). Sample selection bias and presence-only distribution models: implications for background and pseudo-absence data. Ecol Applications.

[44] Ruiz MO, Walker ED, Foster ES, Haramis LD, Kitron UD. Association of West Nile virus illness and urban landscapes in Chicago and Detroit. Int J Health Geogr. 2007;6:10. doi:10.1186/1476-072X-6-10. 10.1186/1476-072X-6-10 PMC1828048 17352825

[45] Rochlin I, Turbow D, Gomez F, Ninivaggi DV, Campbell SR (2011). Predictive mapping of human risk for West Nile Virus (WNV) based on environmental and socioeconomic factors. PLoS ONE.

[46] Gibbs SEJ, Wimberly MC, Madden M, Masour J, Yabsley MJ, Stallnecht DE (2006). Factors affecting the geographic distribution of West Nile virus in Georgia, USA: 2002–2004. Vector Borne Zoonotic Dis.

[47] Saxena R, Das MK, Nagpal BN, Srivastava A, Gupta SK, Kumar A (2014). Identification of risk factors for malaria control by focused interventions in Ranchi district, Jharkhand, India. J Vector Borne Dis..

[48] Moore ID, Gessler PE, Nielsen GA, Peterson GA. Soil attribute prediction using terrain analysis. Soil Sc Soc Am J. 1993;57(2):443–52.

[49] Warren SD, Hohmann MG, Auerswald K, Mitasova H (2004). An evaluation of methods to determine slope using digital elevation data. CATENA.

